# Antifungal Activity of Rue Essential Oil and Commercial Chitosan on Native Corn Foliar Diseases

**DOI:** 10.3390/plants12193416

**Published:** 2023-09-28

**Authors:** Luis Fernando Ceja-Torres, Sigifredo López-Díaz, María Guadalupe Silva-Ramos, José Teodoro Silva-García, José Roberto Medina-Medrano, Germán Fernando Gutiérrez-Hernández

**Affiliations:** 1 Instituto Politécnico Nacional, Centro Interdisciplinario de Investigación para el Desarrollo Integral Regional Unidad Michoacán, Jiquilpan 59510, Michoacán, Mexicolupish88@hotmail.com (M.G.S.-R.);; 2 Licenciatura en Genómica Alimentaria, Universidad de La Ciénega del Estado de Michoacán de Ocampo, Sahuayo 59103, Michoacán, Mexico; 3 Instituto Politécnico Nacional, Unidad Profesional Interdisciplinaria de Biotecnología, Ticomán, Ciudad de México 07340, Mexico; gfgutierrez@ipn.mx

**Keywords:** foliar fungi, corn, *Ruta graveolens*, chitosan

## Abstract

Native corn in Cherán, Michoacán, southwestern Mexico, represents a high-impact economic, social, and religious support, although its yield is low due to fungal diseases. Fungicides are mainly used for their control, but the fungi involved create resistance. The aims of this study are to determine the incidence of foliar diseases in the field, isolate the causal fungi, evaluate the in vitro effect of the essential oil of rue (*Ruta graveolens*) on them, and identify the secondary metabolites. The essential oil was obtained using the steam distillation technique on fresh plants. Also used was an industrial-grade chitosan, and the commercial fungicide benomyl was used as a positive control. Rue essential oil was characterized by mass spectrometry with ultra-high-performance liquid chromatography with electrospray ionization (UHPLC-ESI). The highest incidence of disease was obtained for leaf rust (35%), followed by gray leaf spot (GLS) (24%) and leaf blight (19%). Rue essential oil inhibited 100% of the mycelial growth of *Coniothyrium phyllachorae* and 96% of the mycelium of *Exseroilum turcicum*. The benomyl fungicide effectively inhibited *C. phyllachorae* (86 to 91%), but not *E. turcicum*, with the opposite effect when using chitosan by inhibiting 89 to 90% of the latter’s mycelial development. The majority compound of the essential oil of *R. graveolens* was 2-(3-phenylprop-2-enoyl)chromen-4-one; however, fatty acids were also detected: linoleic, palmitic, and retinoic acid.

## 1. Introduction

Maize is the most cultivated product in Cherán, Michoacán, Mexico, because it is essential for the self-consumption of families throughout the year and also because it generates income by being sold in the same community or in neighboring towns [1]. It is a seasonal crop that is planted in the ‘year and time’ modality, or biennial rotation; however, it is not unaffected by diseases caused by fungi such as leaf blight (*Exseroilum turcicum*), gray leaf spot (GLS) (*Phyllachora maydis*, *Monographella maydis* and *Coniothyrium phyllachorae*), and leaf rust (*Puccinia sorghi*) [2,3].

In some producing states of Mexico, such as Sinaloa and Guerrero, the incidence and severity of these diseases decrease maize yield by 50 to 55% [4,5]. However, there are no reports of the incidence and severity of leaf blight, GLS, and leaf rust in the Purépecha area of Cherán, Michoacán, where conditions may be optimal for their development [6].

Regarding the management of these diseases, chemical control using the fungicides pyraclostrobin, propiconazole, tebuconazole, difenoconazole, epoxyconazole, fluodioxonil, chlorothalonil, fluazinam, mancozeb fluoxastrobin, trifloxystrobin, azoxystrobin, carbendazim, and benomyl has been tested in vitro and in commercial batches both separately and in combination [5,6,7]. But the use of pesticides in Mexico is related to negative effects on terrestrial and coastal ecosystems, the health of agricultural workers and their families, and residues in foods such as milk, vegetables, and grains [8]; however, the greater environmental pressure due to the use of pesticides depends on the different production systems [9].

In the same way, some foliar diseases can be controlled by resistant hybrids [10], but their use is limited in that region because native corn is mostly planted. However, these restrictions could decrease crop yields and, at the same time, food products for an increasing population [11]. Therefore, as a mitigation measure, the use of biofungicides through plant extracts is proposed in this paper, since various studies have shown that they have the capacity to inhibit the growth of some phytopathogenic fungi [12].

The main interest of this work was to use the essential oil of *R. graveolens* as a natural extract to control phytopathogenic fungi in corn. It was subjected to comparison with another natural product, namely, chitosan, since this is a biopolymer whose antifungal capacity has been widely demonstrated to inhibit the mycelial growth of several phytopathogenic fungi as this polymer affects the synthesis of the cell wall and its membrane structure [13,14,15].

Due to the aforementioned factors, the aims of the present work were to determine the incidence and severity of leaf blight, GLS, and leaf rust in the region of Cherán, Michoacán, and to evaluate the antifungal effect of rue essential oil compared with chitosan, a biopolymer with fungicide effects, on *Coniothyrium phyllachorae* and *Exserohilum turcicum*, and to identify the nonvolatile and semi-volatile secondary metabolites of rue essential oil using ultra-high-performance liquid chromatography (UHPLC) since the reported antifungal effect of *Ruta graveolens* is only attributed to volatile compounds.

## 2. Results

### 2.1. Incidence and Severity of Foliar Diseases

**Figure 1:**
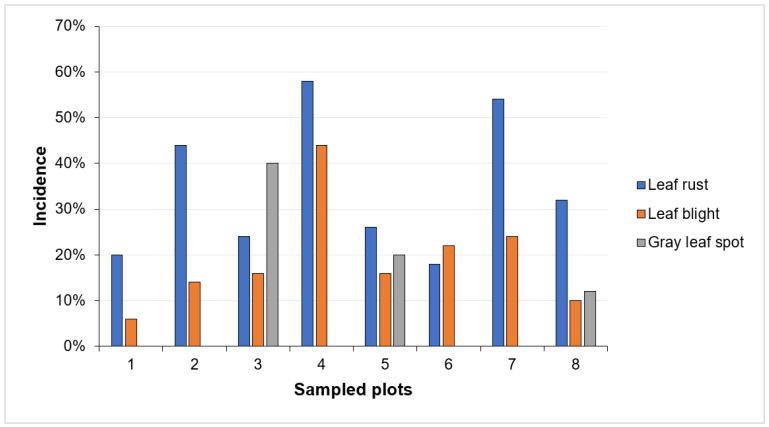
Incidence percentage of foliar diseases on native corn in Cherán, Michoacán, Mexico.

The incidence of leaf blight was 6% to 44% in the sampled plots, with 19% average incidence (Figure 1). The severity of the disease reached grades 3 and 4 according to the scale proposed by Felix-Gastélum et al. [2]. This low incidence in the maize plantations in Cherán, Michoacán, may be because the temperatures are lower than in other maize-growing areas with higher temperatures (18 to 27 °C), and the region has high humidity due to prolonged periods of rain during the growing season [16].

The GLS was found in three of the eight sampled plots, with incidences of 12, 20, and 40% (Figure 1). This disease occurs mainly in cool and humid areas [17], with similar conditions to those where leaf blight also develops. The degree of severity for this disease was 3 and 4 according to the scale proposed by Hernández and Sandoval [18]. It is important to note that the incidence and severity of the GLS reduces corn yields [19].

Another important disease in Cherán, Michoacán, is leaf rust, and three plots were strongly affected with incidences of 44, 54, and 58% with degrees of damage at 3 and 4 according to the Peterson scale [20]. The lowest incidences were registered in five plots, which ranged between 18% and 32% (Figure 1) with degrees of damage at 2 and 3 on the same scale. It should be noted that rust occurs frequently in tropical and temperate climates with high relative humidity [21]. This last characteristic is associated with the plots sampled in Cherán, Michoacán, at altitudes around 2440 masl. Farmers from Cherán, Michoacán, consider that foliar diseases of corn have become economically important due to the irregularity of the rainy season, the delay in the planting season, and climate change.

### 2.2. In Vitro Fungal Inhibition

#### 2.2.1. Percentage of Inhibition of *C. phyllachorae*

**Figure 2:**
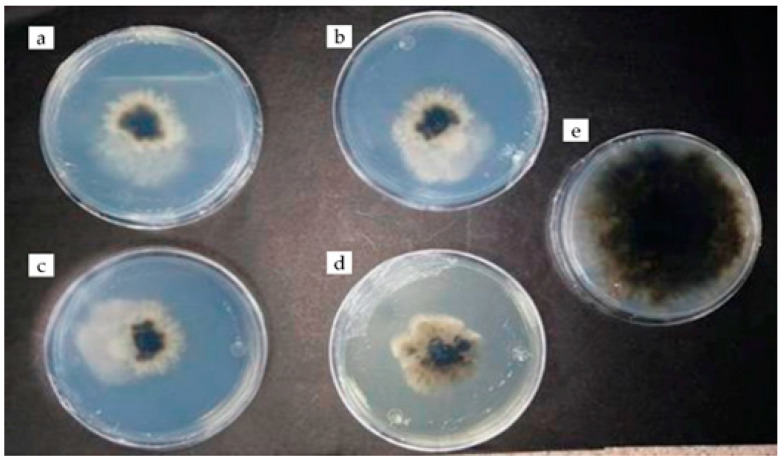
Antifungal activity of chitosan (5 μL) against *Coniothirium phyllachorae* (**a**–**d**) and control fungus (**e**) at 192 h of evaluation.

**Figure 3:**
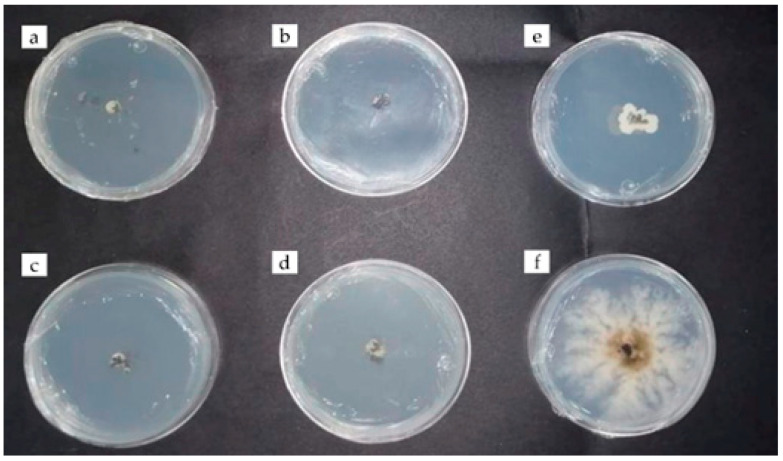
Antifungal activity of rue essential oil (3 μL) against *Coniothirium phyllachorae* (**a**–**d**) with benomyl fungicide (**e**) and control fungus (**f**) at 192 h of evaluation.

Chitosan was less effective in inhibiting the mycelial growth of *C. phyllachorae* (Figure 2) compared to rue essential oil (3 μL), which was 100% effective (Figure 3) from the experiment start to 192 h; the inhibition percentage was higher than that obtained with the benomyl fungicide (91 and 94%) (Table 1). Regarding the effect of the field application of this fungicide, it has been documented that corn grain production can be 5.4 times higher with its application than without [5].

In the laboratory, orange peel essential oil was also 100% effective against *Colletotrichum gloeosporioides*, *Penicillium indicum*, *Fusarium solani*, *Rhizopus stolonifer*, and *Aspergillus flavus*, fungi that affect postharvest papaya fruits [22]. In addition, the essential oils of cinnamon, clove, and oregano leaf and bark inhibited the development of *Penicillium digitatum* in vitro by 90 to 100% [23]. However, no other reports were found on the inhibition of *C. phyllachorae* via the effect of rue essential oil.

#### 2.2.2. Percentage Inhibition of *E. turcicum*

**Figure 4:**
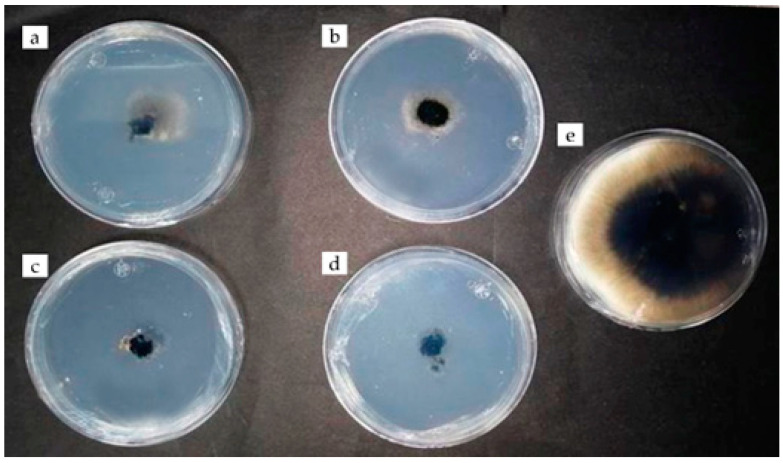
Antifungal activity of chitosan (5 µL) against *Exserohilum turcicum* (**a**–**d**) and control fungus (**e**) at 192 h of evaluation.

**Figure 5:**
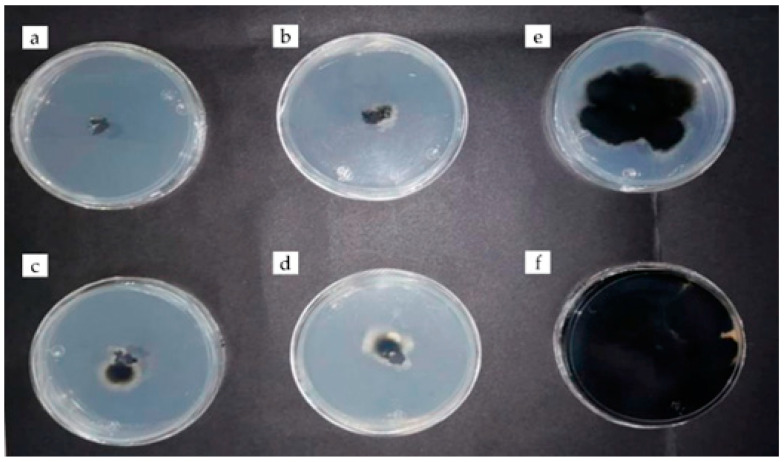
Antifungal activity of rue essential oil (3 µL) against *Exserohilum turcicum* (**a**–**d**) with benomyl fungicide (**e**) and control fungus (**f**) at 192 h of evaluation.

There were no statistically significant differences between the effects of chitosan and rue essential oil, with inhibitions of 96 to 89% (Table 2, Figure 4 and Figure 5). Similar results indicate that *Ruta graveolens* essential oil has powerful antifungal activity against *Aspergillus fumigatus* and *Cladosporium herbarum* [24]. As well as the combined application of chitosan and cinnamon essential oil, the preparations were also effective against fungi isolated from avocado (*Colletotrichum acutatum* and *C. gloeosporioides*) because its mycelial growth was totally reduced [25]. On the other hand, the essential oil of mandarin inhibited the growth of *Penicillium digitatum* in vitro, which causes green mold on citrus fruits in postharvest [26]. Other essential oils extracted from Amazonian plants (*Ocotea quixos* and *Piper adencum*) inhibited the growth of *Aspergillus oryzae*, *Cladosporium cladosporioides*, *Fusarium solani*, *Rhizopus stolonifer*, *Moniliophthora roreri*, and *Phytophthora* sp. by up to 94% average inhibition [27]. It should be noted that no other reports were found on the inhibition of *E. turcicum* due to the effect of rue essential oil.

### 2.3. Secondary Metabolites of Rue Essential Oil

Almost all of the works related to the chemical characterization of rue essential oil refer to volatile compounds analyzed using gas chromatography and gas chromatography–mass spectrometry, and the main components identified were undecanone-2,2-nonanone, and 2-acetoxy tetradecanone. These interesting results also showed the antibacterial activity effect of rue essential oil, and this effect was attributed to volatile components. This conclusion made us look for non-volatile and semi-volatile constituents using the chromatography of ultra-high-resolution liquids with electrospray ionization (UHPLC-ESI). According to the literature, the compounds found using this method also have an important effect against phytopathogenic fungi [28,29].

**Figure 6:**
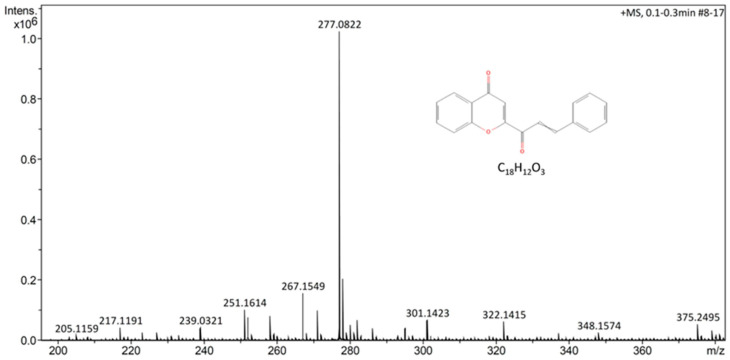
Positive-ion mass fragmentation pattern of semi-volatile cinnamoyl chromone (2-(3-phenylprop-2-enoyl)chromen-4-one) obtained by UHPLC-ESI-MS analyses.

The analysis of the essential oil of *R. graveolens* by UHPLC-ESI indicates that the principal and majority component was the semi-volatile cinnamoyl chromone (IUPAC name: 2-(3-phenylprop-2-enoyl)chromen-4-one) with a mass charge ratio of 277.0831 *m*/*z* (Figure 6); palmitic acid, linoleic acid, and retinoic acid were also present in the essential oil of *R. graveolens* (Table 3). The chemical characterizations of the essential oils of *R. graveolens* that are regularly reported were carried out using gas chromatography and gas chromatography coupled with mass spectrometry (GC–MS), and indicate that the main components are the aliphatic ketones 2-undecanone and 2-nonanone [30,31]. It has also been documented that *Ruta graveolens* is composed of around 69.2% ketones, which have antimicrobial activity and are found in all parts of the rue plant [32]. However, it is important to highlight that the chromone identified using this other methodology contains alkoxy or hydroxyl groups [33], which are molecules that have a wide range of biological activities such as antifungal, antioxidant, antiallergic, antiviral, antihypertensive, anti-inflammatory, antitubulin, and anticancer properties. In addition, the fatty acids linoleic, palmitic and retinoic acid present in the essential oil of *R. graveolens* have demonstrated their activities against plant pathogenic fungi [34,35,36].

## 3. Materials and Methods

### 3.1. Obtaining Rue Plants and Phytopathological Sampling Sites

The rue plants were collected in the town of Cherán, Michoacán, in the Meseta Purépecha region, at the coordinates 19°41′15″ N and 101°57′17″ W, at a median height of 2380 masl [37].

The disease incidence samplings were carried out in eight maize plots, located between 2487 masl (19°40′17.5061″ N, 101°55′57.0813″ W) and 2530 masl (19°43′24.3793″ N, 101°54′53.7448″ W). The five-point sampling scheme was used, considering 100 plants per plot (20 plants in each of the corners and 20 plants in the center). The incidence was calculated using the formula:

Incidence (%) = total diseased plants/total sampled plants × 100(1)

Leaf blight severity (LBS) was scored using a scale of 0 to 4, through the average percentage of affected leaf area, a scale proposed by Felix-Gastélum et al. [2], where 0 = no detectable lesions, 1 = some lesions on leaves (≤5%), 2 = various small and large lesions on many leaves (5.1–10%), 3 = lesions on many leaves (10.1–15%), and 4 = many large lesions (15.1–20%). The severity of the GLS was assessed according to the scale proposed by Hernández-Ramos and Sandoval-Islas [18], made up of seven classes: 0 (0–0), 3 (1–6), 12 (7–22), 38 (23–55), 72 (56–84), 91 (85–95), and 98 (96–100)% of necrotic leaf area. The severity of the rust was evaluated using the scale of Peterson et al. [20], which considers five categories: 1 = 1%, 2 = 5%, 3 = 10%, 4 = 20%, and 5 = 50% of leaf area with the presence of uredospore pustules.

### 3.2. Isolation and Identification of Fungi

From the plots in Cherán, Michoacán, maize leaves with symptoms of leaf blight and GLS were collected. In the Phytopathology Laboratory of the CIIDIR IPN Michoacán Unit, the leaves were washed with water, sectioned into small cube-shaped pieces of approximately 1.5 cm, immersed in a 3% sodium hypochlorite solution for 2 min, and rinsed three times with sterile distilled water. In the laminar flow cabinet, diseased tissues were left to dry on sterile paper towels for 10 min. The leaf sections were then placed on potato dextrose agar (PDA) medium acidified with 85% lactic acid, and incubated at 28 °C for 7 d. The fungi frequently isolated from maize leaves with symptoms of leaf blight and GLS were morphologically identified as *Exserohilum turcicum* and *Coniothyrium phyllachorae*, respectively.

### 3.3. Obtaining Rue Essential Oil

This process began with the washing of rue leaves and stems with running water to remove dust and particle remains. They were then left to dry in the shade at room temperature for 22 days to dehydrate. The extraction of the essential oil was carried out by steam-dragging in a Clevenger-type apparatus following the methodology described by Díaz [29]. For this, 250 g of the dry material was introduced into the balloon flask and 400 mL of water was added for an extraction time of 2 h. The oil obtained was refrigerated in an amber bottle at −70 °C until its use and chemical characterization.

### 3.4. Inhibition of the Growth of Phytopathogenic Fungi In Vitro

Growth inhibition bioassays were made using the direct contact technique proposed by Duarte et al. [38]. In the center of each Petri dish with PDA culture medium, a 5 mm diameter sterile filter paper disk was placed and 3, 5, or 10 μL of the corresponding treatment was added, to which a 4 mm diameter disk with mycelium of the phytopathogenic fungus was placed and incubated at 28 °C until the pathogenic fungus without treatment (control) completely covered the surface of the Petri dish, or until 192 h of incubation. The treatments were chitosan, rue essential oil, and the commercial fungicide benomyl. Four repetitions were performed. The radial growth of each fungus was measured with a ruler, graduated in cm, every 24 h to determine the percentage of inhibition of radial growth using the formula:

Radial growth inhibition (RGI) = (R1 − R2)/R1 × 100(2)

where R1 refers to the radial growth of the pathogen without treatment (control) and R2 to the radial growth of the exposed pathogen with treatment [39].

### 3.5. Preparation of Chitosan Solution

Chitosan was dissolved at 1 percent (*w*/*v*) with 1 percent (*v*/*v*) acetic acid. The pH was adjusted to 5.8 ± 0.02 with 1 N NaOH. To ensure that the chitosan was entirely dissolved in the solution, it was stirred at room temperature overnight.

### 3.6. Preparation of Benomyl 50% PW p/p (500 g/kg)

Next, 1 g of benomyl powder was dissolved in 1000 mL of sterile distilled water. These proportions were according to the manufacturer’s terms of use.

### 3.7. Identification of Secondary Metabolites of Rue Oil

The identification of the secondary metabolites of rue essential oil was carried out following the methodology established by Díaz-Montes et al. [40]. An ultra-high-performance liquid chromatography (UHPLC) system (Thermo Scientific, Waltham, MA, USA) equipped with a photo diode array detector (PDA) was used for the analysis. The chromatographic separation was performed on a Hypersil C18 column (50 mm × 2.1 mm and 1.8 μm particle size) (Thermo Scientific, Waltham, MA, USA). The samples (0.3 μL) were injected into the system. The mobile phases were A: acetonitrile (20%) and B: water–trifluoracetic acid pH 2 (80%) in isocratic conditions, with a flow rate of 104 μL/min for a running time of 9.87 min. The UHPLC system was coupled with a micro TOF-Q mass spectrometer (Bruker Daltonics, Karlsruhe, Germany). The conditions of the micro TOF-Q were as follows: 2.7 kV capillary voltage; ionization source in negative electrospray mode (ESI^−^ and ESI^+^); scan range, *m*/*z* 50–3000; set collision cell RF, 200.0 Vpp; set nebulizer, 0.4 bar; desolvation gas flow set at 4.0 L/min; and source temperature, 180 °C. The mass data were processed using Bruker Compass Data Analysis version 4.1 software (Bruker Datonics, Karlsruhe, Germany).

### 3.8. Data Analysis

A completely randomized design with four repetitions was used. The percentages of the inhibition of radial growth were transformed using arcsine √x/100 and subjected to an analysis of variance (ANOVA) and Tukey’s test (*p* < 0.05). These analyses were carried out using SAS software version 9.0 (SAS Institute Inc., Cary, NC, USA).

## 4. Conclusions

Native corn from Cherán, Michoacán, Mexico, is affected by leaf rust, GLS, and leaf blight, with average incidences of 35, 24, and 19%, respectively. Rue essential oil inhibited the radial growth of *Coniothyrium phyllachorae* (100%) and *Exserohilum turcicum* (96%) in vitro. Chitosan inhibited *E. turcicum* by 89 and 90%, respectively. The majority constituent of the essential oil of *R. graveolens*, obtained from the Meseta Purépecha region, was 2-(3-phenylprop-2-enoyl)chromen-4-one (cinnamoyl chromone).

## Figures and Tables

**Table 1:** Antifungal activity of commercial chitosan and rue essential oil against the fungus *Coniothyrium phyllachorae*.

	Percentage of Inhibition (%)							
Treatments	24 h	48 h	72 h	96 h	120 h	144 h	168 h	192 h
Benomyl (5 μL)	100 ± 0.0 ^a^	100 ± 0.0 ^a^	100 ± 0.0 ^a^	100 ± 0.0 ^a^	100 ± 0.0 ^a^	98 ± 2.3 ^a^	95 ± 1.5 ^a^	91 ± 3.7 ^a^
Benomyl (10 μL)	100 ± 0.0 ^a^	100 ± 0.0 ^a^	100 ± 0.0 ^a^	100 ± 0.0 ^a^	100 ± 0.0 ^a^	100 ± 0.0 ^a^	99 ± 1.5 ^a^	94 ± 2.4 ^a^
Chitosan (5 μL)	94 ± 7.3 ^a^	73 ± 11.0 ^b^	65 ± 6.7 ^b^	60 ± 3.8 ^b^	57 ± 3.8 ^b^	48 ± 4.7 ^b^	43 ± 5.1 ^b^	37 ± 5.5 ^b^
Chitosan (10 μL)	100 ± 0.0 ^a^	73 ± 9.9 ^b^	65 ± 8.4 ^b^	43 ± 4.0 ^c^	41 ± 5.2 ^c^	38 ± 6.5 ^c^	30 ± 4.9 ^c^	25 ± 2.0 ^c^
Rue essential oil (3 μL)	100 ± 0.0 ^a^	100 ± 0.0 ^a^	100 ± 0.0 ^a^	100 ± 0.0 ^a^	100 ± 0.0 ^a^	100 ± 0.0 ^a^	100 ± 0.0 ^a^	100 ± 0.0 ^a^

Values are expressed as mean ± standard deviation of four repetitions. Different letters in the same column indicate significant differences (Tukey, *p* < 0.05).

**Table 2:** Antifungal activity of commercial chitosan and rue essential oil against the fungus *Exserohilum turcicum*.

	Percentage of Inhibition (%)							
Treatments	24 h	48 h	72 h	96 h	120 h	144 h	168 h	192 h
Benomyl (5 μL)	75 ± 10.3 ^b^	49 ± 5.7 ^c^	27 ± 3.2 ^c^	29 ± 3.8 ^c^	19 ± 2.4 ^d^	18 ± 2.4 ^d^	17 ± 2.0 ^c^	16 ± 3.5 ^c^
Benomyl (10 μL)	88 ± 10.2 ^a^	84 ± 4.0 ^b^	74 ± 6.0 ^b^	63 ± 9.8 ^b^	56 ± 4.2 ^c^	46 ± 3.5 ^c^	37 ± 3.8 ^b^	35 ± 5.5 ^b^
Chitosan (5 μL)	100 ± 0.0 ^a^	94 ± 4.0 ^a^	93 ± 4.5 ^a^	96 ± 3.0 ^a^	95 ± 2.0 ^ab^	92 ± 1.5 ^b^	91 ± 2.0 ^a^	89 ± 6.1 ^a^
Chitosan (10 μL)	100 ± 0.0 ^a^	100 ± 0.0 ^a^	96 ± 4.6 ^a^	96 ± 2.5 ^a^	96 ± 3.2 ^ab^	94 ± 2.0 ^ab^	91 ± 2.0 ^a^	88 ± 4.0 ^a^
Rue essential oil (3 μL)	100 ± 0.0 ^a^	100 ± 0.0 ^a^	100 ± 0.0 ^a^	100 ± 0.0 ^a^	100 ± 0.0 ^a^	98 ± 2.8 ^a^	96 ± 1.1 ^a^	96 ± 2.4 ^a^

Values are expressed as mean ± standard deviation of four repetitions. Different letters in the same column indicate significant differences (Tukey, *p* < 0.05).

**Table 3:** Main non-volatile and semi-volatile components of essential oil of *R. graveolens*.

Molecule	Mass Charge Ratio	Volatility
Cinnamoyl chromone *	277.0831 *m*/*z*	Semi-volatile
Palmitic acid	255.2318 *m*/*z*	Non-volatile
Linoleic acid	277.2165 *m*/*z*	Non-volatile
Retinoic acid	299.2012 *m*/*z*	Non-volatile

* IUPAC name: 2-(3-phenylprop-2-enoyl)chromen-4-one.

## Data Availability

The authors declare that the data supporting the findings of this study are available within the article.
